# Secure routing in the Internet of Things (IoT) with intrusion detection capability based on software-defined networking (SDN) and Machine Learning techniques

**DOI:** 10.1038/s41598-023-44764-6

**Published:** 2023-10-21

**Authors:** Kunkun Rui, Hongzhi Pan, Sheng Shu

**Affiliations:** 1 School of Information and Artificial Intelligence, Anhui Business College, Wuhu, AnHui 241002 China; 2 College of Industrial Education, Technological University of the Philippines, 0900 Manila, Philippines

**Keywords:** Computational science, Computer science

## Abstract

Routing and security are the two main prerequisites for ensuring the correct operation of wireless networks. The importance of these cases doubles in wide networks such as IoT. This paper presents an algorithm to improve Secure Routing in IoT called SRAIOT. This algorithm uses a hierarchical structure to determine the connections between network components and data transfer routing. In SRAIOT, the network structure is managed hierarchically and through SDN. For this purpose, the IoT network is first divided into a set of subnets using the SDN solution, communication control and authentication are managed using the controller nodes of each subnet. The communication between two objects (located in different subnets) will be possible if their identity is confirmed through the controller nodes related to them. On the other hand, in order to identify the sources of attacks and network security threats, the controller nodes in each subnet monitor the network traffic pattern using an ensemble learning model and identify possible attacks in their subnet. The performance of SRAIOT was tested in the simulation, and the results were compared with previous methods. The results of these tests show that SRAIOT improves network performance regarding routing and detecting attacks*.*

## Introduction

IoT is one of the new technologies in the field of intelligent communication, and its advantages cause various organizations and institutions to join this technology every day. Based on the studies, the future of the communication world can be imagined in the context of the IoT^1^. This feature has caused researchers to be interested in implementing the IoT based on new technologies (such as the 5G network)^2^.

One of the key aspects of the introduction of IoT is the ability to support many devices compared to the current number. Communication management between billions of connected sensors and radio devices is one of the new envisioned applications for the IoT^3–5^. Providing a communication platform for this number of equipment will cause new security issues. For example, in such a network, cyber-attack victims may lose access to equipment in their homes, cars, or mobile phones^6^. For this reason, in various research works, solutions have been provided to ensure communication security in these networks^7,8^. Still, it remains an unsolved problem, and this network will need more efficient security solutions.

With the significant increase in the number of connected devices and applications in IoT, this environment provides more opportunities for attackers to execute threats such as Denial of Service (DoS) attacks^9^.

In this situation, two-way authentication between users of this network should be done more efficiently than in previous generations to prevent impersonation or man-in-the-middle attacks^10^. For this reason, it is inevitable to provide a fast, accurate, and resistant security solution to secure communications in IoT.

On the other hand, based on recent studies, more than the use of encryption solutions for coding IoT communications and data is needed. Because in addition to not being able to detect the attacker, it causes a waste of network computing resource ^11^. Routing is one of the basic operations in communication networks, which, in addition to efficiency, must be done securely. The requirement for providing security in data routing of the IoT, is to have an efficient framework for organizing network devices and also to use a precise approach in order to identify possible attacks through the processing of huge amounts of network data. Due to issues such as the heterogeneity, limited computational capabilities and the lack of full reliability, the mentioned security processes cannot be fully assigned to things. On the other hand, the conventional centralized security systems are not efficient enough to secure communications in huge networks such as IoT due to high overhead. These issues cause the need to review security strategies for routing in the Internet of Things.

The need for an integrated and efficient strategy for secure routing and simultaneous detection of attacks in the IoT has motivated the current research. Based on this motivation, the main objective of the current research has been to provide a comprehensive and efficient approach for secure routing with the ability to detect attacks in IoT, which can overcome the shortcomings of previous solutions in dealing with attacks. This research pursues the main goal mentioned in two minor objectives: First, providing a new solution for decomposing the network into sub-networks, based on which the processes related to authentication, routing and monitoring of network communications can be performed more efficiently. Second, providing an accurate model for detecting intrusions that is highly accurate and fully compatible with the limited capabilities of IoT equipment.

This research uses SDN as a solution to fulfill the first objective. Therefore, using a novel SDN-based network decomposition mechanism, the complex problem of securing communications in a very large network is turned into several problems of securing communications in smaller subnets. Decomposing the network into a set of subnets by the proposed strategy can be useful in solving two major problems. First, this strategy can improve routing efficiency for large networks by reducing major congestion and more balanced load distribution. Secondly, by using this strategy, the problem of network security monitoring can be broken down into several simple sub-problems implicitly, the result of which will be to prevent the burden on third-party monitoring nodes and reduce the overhead and delay caused by monitoring processes. The proposed method carries out the mentioned process based on the prediction of the links durability, which makes the formed structure able to have a good performance in dynamic conditions. Also, ensemble learning is used as a solution for the second objective. This hybrid model uses a new combination of classifiers which is effective in improving the detection accuracy. In this ensemble model, the combination created through learning models can cover the minor error of each classifier, which will result in achieving an intrusion detection system with higher accuracy compared to the case that each classifier is used separately. The novel combination of the above two strategies has not been studied in previous similar studies and distinguished the current research from previous works.

The contribution of this article can be summarized as follows:

- In this research, a hierarchical framework based on SDN is presented to improve network users' authentication process. The proposed solution first divides the IoT network into a set of subnets using the SDN architecture, and communication control and authentication are performed using the controller nodes of each subnet. Communication between two things (located in different subnets) will be possible if their identity is confirmed through the controller nodes related to them.
- SRAIOT includes an intrusion detection system that identifies types and sources of attacks before any malicious activity occurs. For this purpose, the controller nodes in each SDN subnet control the traffic pattern of the network by using an intrusion detection model based on machine learning techniques and identify possible attacks in their subnet. This intrusion detection system is a new combination of classification models based on machine learning, which maximizes the accuracy of attack detection by using the majority voting strategy between the output of the classifiers.

The remainder of the paper is organized as follows: The second part reviews the previous related studies in the field of secure routing in the IoT. In the third part, SRAIOT is explained, and in the fourth part, the efficiency of SRAIOT is evaluated from different aspects. Finally, in the fifth section, the findings are summarized.

## Literature review

Routing and security are among the most interesting research areas in IoT networks. Although the number of studies that follow these two fields simultaneously is more limited; However, the efforts made in this field are significant. In this section, some recent research works in this field are studied. Previous researches in this field can be divided into two general categories. The first group of methods focused on secure routing in the Internet of Things. While the methods of second category have focused on analyzing network traffic and detecting intrusions in it.

### Secure routing in IoT

The purpose of this type of research is to provide strategies for determining data transmission paths without involving nodes that may threaten the security of other nodes or the information being exchanged. These methods often use criteria such as node reputation, or a set of rules (which may be automatically generated through machine learning techniques) to determine which nodes participate in routing. Also, the use of encryption techniques is considered a popular strategy in this category of methods. In^12^, a cryptographically based secure routing algorithm for smart health network with IoT infrastructure is presented. This method, called cross-layer and cryptography-based secure routing (CLCSR), pursues goals such as attack detection, privacy protection, and secure data exchange through two phases: the goal of the first phase is network clustering, in which a probabilistic model is used to determine network security risks and also select the cluster head at the same time. In the second phase, a Lightweight Encryption Algorithm (LEA) was used to provide users' privacy and secure data transmission. This approach does not provide an efficient method to prevent malicious nodes from being selected as cluster head.

In^13^, deep learning techniques are used for secure routing in the 5G wireless sensor network based on the IoT. In this research, deep convolutional neural network (DCNN) and distributed particle filtering evaluation scheme (DPFES) have been used in order to form an attack detection system with multiple agents. In this system, the position of monitoring nodes is determined to detect attacks through DPFES, and in each determined position, a DCNN model is deployed to monitor the performance of things and detect attacks. This solution is more focused on the aspect of network security and the routing strategy used in this research does not have a significant advantage over recent methods. Also, the centralized DCNN model is computationally expensive which makes is hard to implement on big networks.

In^14^, a framework called AROSTEV is presented for secure routing in the structure of the IoT. This framework provides the possibility of detecting rank distortion attacks in routing through three techniques. For this purpose, in this article, rank distortion attacks are classified into three categories: decrease, increase, and inconsistency of the rank, and a separate technique is presented to detect each of them. This method requires a hop-by-hop analysis of each route for acceptable protection, which creates a significant processing load in wide networks in addition to high communication overhead.

In^15^, a credit-based algorithm for secure routing in the IoT using optimization strategies is presented. This research presented a new optimization strategy called rider foraging optimization (RFO), which is a combination of two optimization algorithms,rider optimization algorithm (ROA) and bacterial foraging optimization (BFO), and its task is to discover the optimal routes between any two nodes in the network. In this method, the Low-energy adaptive clustering hierarchy algorithm (LEACH) is used to cluster the network structure. Also, the determination of the optimal route is done based on the validity, energy, and delay criteria. The method presented in^16^ also uses an improved version of the Blowfish algorithm in a similar way to determine secure data transmission routes in a wireless sensor network based on the IoT. The clustering algorithm used in these methods is not energy efficient and does not consider security measures for selecting cluster heads, which can lead to network vulnerability.

In^17^, the combination of SDN and Blockchain technology has been used to provide routing security in the IoT. In this method, the network structure is divided into a set of domains, and each subdomain is monitored by a controller. It should be noted that this method does not provide a solution for managing unstable network connections and uses all available links for data exchange. To ensure the security of network communications, a Blockchain-based architecture is used, which enables secure data routing between multiple network domains. In this architecture, all SDN controllers are equipped with Blockchain, and each controller node deploys its domain topology in Blockchain through a smart contract. Although this process involves a high cost, it provides the possibility of faster access to the global topology of the network.

Research in^18^ presented a secure routing algorithm in the IoT based on the smart determination of object validity using a meta-heuristic strategy. In this paper, the chaotic bumble bees mating optimization (CBBMO) algorithm is used for secure data transmission. The CBBMO algorithm is an improved version of the BBMO algorithm, combined with the concepts of chaos theory for faster convergence. This method uses a credit criterion to ensure the security of data transmission routes, which is a combination of direct and indirect credit of the node, and based on that, it identifies the attacking nodes. In this way, the CBBMO algorithm determines the most secure path for data transmission based on the credit values of the intermediate nodes of the existing paths. The requirement for implementing this algorithm in equipment with limited computing power is to reduce the search cycle and population size of the CBBMO, in which case the probability of getting trapped in the local optimum will increase.

In^19^, a secure and opportunistic routing strategy for the IoT based on node intimacy and credit criteria was presented. The purpose of this method is to solve the problem of unbalanced transmission efficiency and security in the message delivery process. In this solution, the criteria of intimacy and credit of nodes are calculated according to the records of encounters with the node and its performance in sending messages. The method presented in^20^ is also a multi-level security protocol inspired by nature for aggregation and routing in the IoT. This method uses the method of node behavior detection to calculate the credit of things and also to determine the pattern of data aggregation. The routing algorithm introduced in this research also includes three main steps: first, the network is segmented into two inner and outer regions based on the position of the nodes, and clusters are formed in each region. In the second step, the data transmission paths between the cluster heads and the data center are secured using a secret key sharing strategy. In the third step, data links are analyzed to minimize the possibility of intrusion. This method has not provided a solution for the safe selection of cluster heads, and if an invalid node is selected as a cluster head, the connection with the entire cluster will be lost.

In^21^, a secure routing algorithm for IoT based on sequence number was presented, which uses the current structure of IoT packets. The objective of this paper is to maximize the packet delivery ratio and network lifetime to improve network performance. This research did not investigate the performance of the method in scenarios such as presence of malicious nodes and was limited only to evaluating the packet delivery rate.

The method presented in^22^ is also a secure and credit-based routing algorithm in the IoT, main goal of this algorithm is to deal with signal processing attacks. This method includes two techniques: first, the combination of fuzzy logic and particle swarm optimization (PSO) algorithm is used to determine secure routes. Then, the encryption technique based on Learning with Errors over Rings (R-LWE) is used for data conversion and providing privacy. In this method, the credit of things is estimated using a computational model and based on the calculated credit, invalid nodes are identified to be ignored in the next cycle of route selection. However, this credit assessment method will lead to an increase in false alarm rate, in unstable communications and noisy environments. Research in^23^, presented a model for improving the security of IoT networks in confronting man-in-the-middle attacks using SDN. This research used deep packet inspection with SDN for traffic separation.

### Intrusion detection systems in IoT

A wide range of researches have focused only on the problem of detecting attacks in IoT. Detection of attacks in computer networks is done through intrusion detection systems and using strategies such as analysis of stateful protocols, statistical anomaly or signature^24^. In general, the role of an IDS is only to detect the existence of an attack and report it^25^. According to recent researches, methods based on machine learning have had a better performance in detecting attacks. Research in^26^, presented a hybrid deep learning-based IDS for IoT networks. In this IDS model, a LSTM is used for extracting features and then, a CNN model is trained based on the extracted features for detecting intrusions in the network. In^27^, an anomaly-based intrusion detection system for Internet of Things is presented, which uses deep learning techniques. This intrusion detection system uses a centralized architecture and needs to monitor all incoming and outgoing network traffic using a convolutional neural network. In^28^, a model based on convolutional neural network for extracting network traffic features and a feature selection model based on Aquila optimizer optimization algorithm are proposed so that attacks in IoT communications can be detected based on them. Both of these methods use centralized architecture for intrusion detection which are not cost-effective for use in large-scale networks. The studied works have been summarized in Table 1.

**Table 1:** Summary of the related works.

References	Year	Research method	Limitation(s)
Kore et al. ^12^	2022	Cross-layer and cryptography-based secure routing using clustering and encryption	Risk of selecting malicious nodes as cluster heads
Rajasoundaran et al. ^13^	2022	Secure routing in the 5G WSNs using DCNN and DPFES	Attack detection model is centralized and computationally expensive
Stephen et al. ^14^	2022	Rule-based detection of rank distortion attacks in IoT	High processing load and communication overhead in large networks
Amit et al. ^15^	2022	Reputation-based secure routing in IoT using RFO	Risk of selecting malicious nodes as cluster heads
Alotaibi ^16^	2021	Secure routing in IoT using Blowfish algorithm	Risk of selecting malicious nodes as cluster heads
Zeng et al. ^17^	2022	Secure routing in IoT using SDN and Blockchain	Not providing a solution for managing unstable links, computationally expensive
Gali et al. ^18^	2022	Secure routing in IoT using CBBMO and chaos theory	Possibly trapping in local optimum for routing through nodes with limited computing power
Yu et al. ^19^	2022	Secure opportunistic routing for IoT based on node intimacy and reputation	High false alarm rate in noisy environments
Chandnani & Khairnar ^20^	2022	Multi-level security protocol for IoT based on clustering and encryption	Risk of network partitioning in case of selecting malicious nodes as cluster heads
Kothandaraman et al. ^21^	2021	Secure routing algorithm in IoT based on sequence number	Evaluation is limited to packet delivery rate
Ragesh & Kumar ^22^	2022	Secure reputation-based routing in IoT using fuzzy logic, PSO and R-LWE	High false alarm rate in noisy environments

## Proposed secure routing algorithm in the IoT (SRAIOT)

This section details the SRAIOT to improve communication security in the IoT structure. In SRAIOT, SDN creates a secure communication platform between network things. In this case, the network structure is divided into a set of subnets. The members of each subnet will be highly similar in terms of position and movement pattern, and this guarantees the stability of network topology communication. Also, in this structure, the task of authenticating and managing the communication of the members of each subnet is assigned to a controller node. In addition to this communication structure, a neural network based learning model is used to monitor network traffic. In this way, each controller node uses this learning model to identify attacks and security threats in its subnet. The assumptions used in SRAIOT are as follows:

- Due to the different technologies for making radio equipment in wireless networks, network nodes have non-homogeneous communication characteristics. As a result, the assumed network is inhomogeneous.
- The assumed network structure is designed based on the 5G network technology; Therefore, it has all the characteristics and requirements of this communication technology.
- The distance between two nodes can be calculated by estimating the strength of the radio signal received by each node. Therefore, if the network equipment does not have a global positioning system (GPS), they can estimate the distance to each other by checking the received signal strength of the adjacent nodes.
- Each controller node in the SDN is equipped with a learning model that can record and process data traffic. This learning model is an artificial neural network; It is used to identify attacks and security threats in the subnet corresponding to the controller node.

SRAIOT to improve communication security in the structure of IoT based on SDN and EL includes the following steps:

1. Formation of network clustering structure based on SDN
2. Formation of network hierarchical tree topology
3. Data routing using a formed structure
4. Detection of attacks based on EL

**Figure 1:**
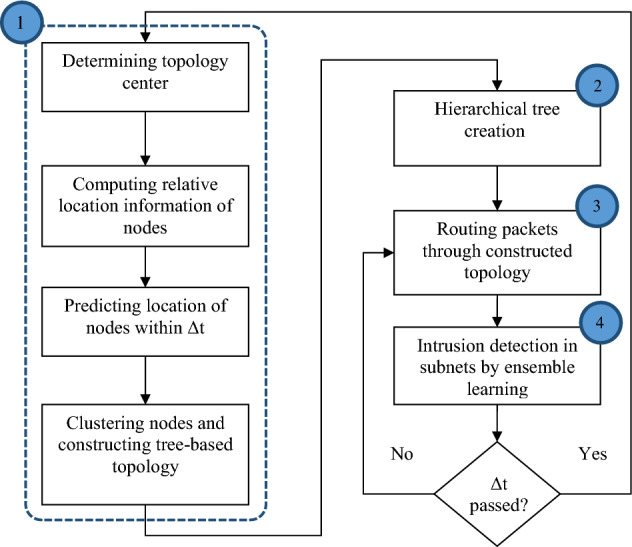
Diagram of the SRAIOT.

The details of SRAIOT steps are shown as a diagram in Fig. 1. As seen in this figure; SRAIOT is repeated in specific time intervals such as Δt. In the first step of SRAIOT, the SDN domain is divided into several subdomains using a clustering solution based on the movement pattern of active nodes, and each part is equipped with a controller to exchange security rules with other subdomains.

In SRAIOT, each controller will provide the list of authenticated users related to its subdomain to other controllers. In this way, if there is a need to establish communication between two users, the user's credit is done by exchanging messages between the controllers. If each of the two sides of the communication is authenticated by at least one controller, the data routing will be done.

To control the network topology, the minimum spanning tree and Prim algorithm are used. In this step, each node forms the topology of the network locally through the construction of minimum-spanning trees. Then, by leveling network nodes and determining the weight of network connections, a hierarchical tree is formed for data routing. Finally, the data is routed to the destination through the hierarchical tree structure. Based on the structure proposed in this research, all the traffic of nodes related to a subnet is exchanged through the controller node of that subnet. Therefore, each controller node continuously uses an EL learning model to analyze network traffic information and identify attacks. This model is composed of three learning models and, based on the statistical information extracted from each traffic flow, identifies the possible presence of attacks in it. Each of these steps is explained in the following.

### Formation of network clustering structure based on SDN

In the first step of SRAIOT, a topology structure will be created to determine the secure communication infrastructure between network things. For this purpose, it is necessary to first identify the list of neighbors of each active node in the network, which is done by exchanging Hello control packets. In this process, each node stores its unique identifier in the content of the control packet, and then by broadcasting this message, it informs its existence to the neighboring nodes. Each active node, upon receiving this message, will add the ID of the sending node to its neighbors list. During these exchanges, the signal strength received from each adjacent node is also measured and recorded by the node. By repeating this process, each active node will produce a list containing the ID of its neighbors as well as the strength of the signal received from them.

In the next step, the network nodes exchange their neighbors lists so that the low-quality network connections are identified and removed. For this purpose, each active node will send the received signal strength from neighboring nodes to them. The received signal strength of node B is shown by node A as RSSI_A,B_. By exchanging the signal strength values, each of the nodes A and B will be informed of the signal strength level received by the other node. In such a situation, a node like A evaluates the quality of its connection with node B based on the following conditions:

- Having the strength of the received signal in the connection between A and B, active node A calculates the average signal power of both sides of the connection ${R}_{AVG}=\frac{RSS{I}_{AB}+RSS{I}_{BA}}{2}$. With this method, the destructive effect of noise in signal evaluation can be reduced to some extent.
- If the average signal strength, ${R}_{AVG}$, is greater than the threshold, P, then the connection between two nodes A and B has sufficient quality and will be considered as an active connection. Otherwise, the connection between the two nodes will be ignored.
- If the connection between A and B does not have the required quality, then the active nodes A and B remove each other from the list of their neighbors.

Implementing this process by each network node establishes a set of communication links with appropriate quality between the active network nodes. Each active node in the network will send its characteristics including ID, position information, and radio range to the active nodes located in its neighborhood using a control packet. Upon receipt of the topology construction control packet by each neighboring node, this information is sent to the neighbor with the highest degree of neighborliness (the node with the highest number of connections). If this process is repeated, the topology construction control packets are sent to the node with the highest degree of neighborhood. This node is called the central node Ct. After receiving all control packets of the topology construction by the central node, a view of the communication pattern of the network nodes will be created by the central node and this node will be able to create the graph of network active nodes. The central node, by using the positional information received from the active nodes, calculates the stability of the connection between both active nodes, such as i and j, as follows^29^:

1 $${T}_{ij}= \frac{d.\mathrm{cos}({\varphi }_{ij})+ \sqrt{{r}^{2}-{d}^{2}{\mathrm{sin}}^{2}({\varphi }_{ij})}]}{{v}_{ij}}$$

$${v}_{ij}=\sqrt{{\left({v}_{i}\mathrm{cos}\left({\varphi }_{i}\right)-{v}_{j}\mathrm{cos}\left({\varphi }_{j}\right)\right)}^{2}+{\left({v}_{i}\mathrm{sin}\left({\varphi }_{i}\right)-{v}_{j}\mathrm{sin}\left({\varphi }_{j}\right)\right)}^{2}}$$

$${\varphi }_{ij}={\mathrm{tan}}^{-1}\frac{{v}_{i}\mathrm{sin}\left({\varphi }_{i}\right)-{v}_{j}\mathrm{sin}\left({\varphi }_{j}\right)}{{v}_{i}\mathrm{cos}\left({\varphi }_{i}\right)-{v}_{j}\mathrm{cos}\left({\varphi }_{j}\right)}$$

In (1), ${v}_{i}$ represents the movement speed of node i, and ${\varphi }_{i}$ specifies the movement angle of this node. Also, r represents the radio range of the node and d represents the distance between two nodes i and j, estimated by sampling the received signal strength. By using the above relations, it is possible to predict whether two nodes i and j will be neighbors after the time interval $\Delta t$ or not. This will happen if ${T}_{neighbor}\ge \Delta t$**.**

By calculating the value of ${T}_{ij}$ for each pair of nodes in the network, a similarity matrix is formed. This matrix contains the movement patterns similarity degree of both pairs of nodes. All the nodes send their estimated communication stability value to the central node Ct so that the topology construction is done. To construct the network topology, the central node integrates the received ${T}_{ij}$ values and categorizes the nodes into clusters using two basic rules. In this method, the nodes that have the same movement pattern are placed in a cluster. To detect the similarity of the movement pattern of two nodes, the following conditions are checked:

- Two nodes should be in the same radio range (both nodes have one-step and direct access to each other)
- It should be predicted that after a period of time $\Delta t$, the distance between two nodes does not exceed the minimum radio range of two nodes.

For the second condition, the method of predicting the position and durability of the connection between two nodes is used (1), and based on these criteria, the information of the movement pattern of users is stored in a matrix like T. The clustering of network nodes is done based on this matrix. Using these two rules, the steps of clustering nodes in the network are as follows:

**Table:** 

Input: < user list L, connection period matrix T >
Output: network clusters C
1. Repeat the following steps until a node is in the list L
2. Pick a random node like x in list L and remove it from L and create a new cluster in C
3. For each node like $y\in L$: if y is a neighbor of x and based on the matrix T, and ${T}_{xy}\ge \Delta t$ then add y to the current cluster in clustering C and omit node y from the list L
4. If L = ϕ, terminate the algorithm otherwise go to step 1

After doing these steps, all network nodes are placed in clusters according to their movement pattern. The next step of SRAIOT is to select the cluster head as the SDN controller. For this purpose, the node that has the highest degree of neighborhood in each cluster is determined as the head of the cluster and the SDN controller. Then, each cluster member node in the network has a direct connection only with its SDN controller (it will not even connect with its neighbors). The goal is to require network users to be authenticated through the SDN controller in order to avoid security risks inside or outside the clusters. Also, by using this structure, each node is required to exchange its traffic with others through the controller node, and thus, it will be possible to monitor this information and detect attacks using the learning model for all information exchanged in the network. After determining the SDN controller as the cluster head, each controller will find the shortest path to the central node Ct through intermediate nodes (which will act as cluster gateways). This process is explained below.

### Formation of network hierarchical tree topology

In this step, the clustered structure of the network in the previous step will be transformed into a hierarchical structure. For this purpose, construction of a hierarchical topology begins with the use of a controller node as a central one. This central node is considered as the root of the hierarchical tree. Therefore, the first step in constructing a hierarchical tree topology is to determine a node as the central node of the network topology. The feature of neighborhood degree can be a suitable feature to determine the topology center. In SRAIOT, first the controller nodes determined in the previous step identify their neighbors by broadcasting all the control packets. Each network node waits for a short time after redistributing the topology construction packet to receive all response packets. Then it informs the neighbors about the number of neighbors by sending multicast packets. By repeating this process, the controller with the largest number of neighbors in the network will be defined and this controller node will be determined as the topology center. During this process, each responding node stores the control message, its information: congestion, energy, and estimated distance in the response packet and sends it to the sender node. This information will be used to weight network connections so that a hierarchical topology with the most suitable features can be produced. The proposed algorithm, based on the information of congestion, distance, and energy of the node, weights the network connections to construct the most suitable hierarchical tree.

In SRAIOT, considering the congestion degree parameter in addition to node energy, the weight of network connections is determined. The purpose of constructing a hierarchical tree based on these weighted connections is to avoid sending data to nodes that are in a congested state and also to provide the possibility of using nodes with higher energy and lower degree of congestion. The formula for calculating the weight of each connection to node i in SRAIOT is as follows:

2 $${W}_{ij}=\left(\frac{{C}_{j} \times {D}_{j}}{{E}_{j}}\right)$$

where Cj is the degree of congestion of child node j, which is calculated by (3).

3 $${C}_{i}=\frac{{T}_{service}}{{T}_{arrival}}$$

Also, ${D}_{j}$ is the estimated distance between the current node and neighboring node j, and ${E}_{j}$ represents the remaining energy of node j. Each node responding to the control message puts the above parameters on its ACK packets and sends them to the sender node. Also, all the values of ${C}_{j}$, ${D}_{j}$, and ${E}_{j}$ parameters are normalized by following equation before using in (2).

4 $${N}_{i}=\frac{{n}_{i}-{n}_{min}}{{n}_{max}- {n}_{min}}$$

As mentioned, the advantage of using this method is to prevent congestion in a node by choosing routes with less congestion and more energy. After determining the weight of all connections by (2), a hierarchical tree structure will be constructed.

After determining the weight of network connections, the central controller node will have the weight of all connections and the list of all network clusters. The shortest paths between the central node and other network clusters construct the hierarchical tree structure. In this way, each controller node (cluster manager) finds the shortest path (the path with the lowest total connection weight) to the central node through intermediate nodes (which act as cluster gateways). In this way, the clustering structure of the network will be transformed into a hierarchical tree topology, which will be used for the secure data routing process in the time period $\Delta t$.

### Data routing using the constructed structure

**Figure 2:**
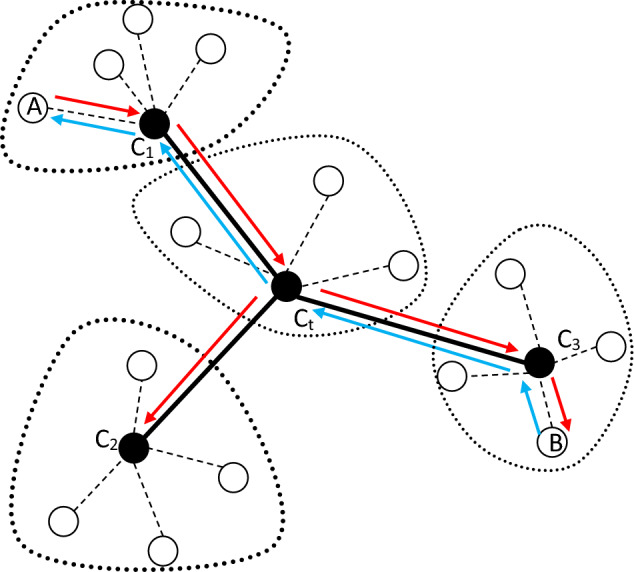
An example of the data routing process in SRAIOT.

After constructing the hierarchical tree topology, this structure will be used for secure data routing. According to the tree topology, it is clear that there is only one path between both subdomains. However, for secure data routing between mobile nodes in the network, the controllers of each subdomain must exchange their members' information. In this way, if a node intends to send data to another node, the source node first sends the ID of the destination node to its subdomain controller. If the destination node is located in the same subdomain, the connection between the two nodes is done by sending a response message to the source node. Otherwise, the controller node sends the message sent from the source to the central controller C_t_. After receiving this message, C_t_ sends packets containing the ID of the destination node to the controllers of other subdomains. The controller that has the destination node in its subdomain sends a confirmation message to the source node through the central node C_t_. In this way, the connection between the two nodes will be established. An example of the routing process in the proposed algorithm is shown in Fig. 2. To keep the simplicity, in this figure, the communications between the gateway nodes are not considered.

In Fig. 2, it is assumed that a node like A in subdomain 1 intends to connect to node B in subdomain 3. In this case, node A first sends a message containing the ID of the destination node to controller C1. Considering that node B is not in the sub-domain of C1, so this controller sends the received packet to controller Ct. This controller also sends this message to other controllers (C2 and C3). Considering that destination node B is located in the subdomain corresponding to node C3, a response packet is sent by this subdomain to the source node. In the end, the data packet is exchanged between two nodes through the discovered path. During data routing by the controller nodes, the process of traffic information analysis and intrusion detection is done using EL. In the following, the structure of the proposed learning model is explained.

### Intrusion detection in each subnet based on EL

**Figure 3:**
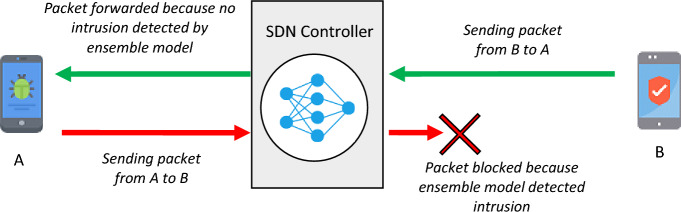
The performance of controller nodes to identify attacks based on EL.

As mentioned, each controller node in the software-based network is equipped with an EL model that can record and process the flowing data traffic by itself. This learning model, which actually consists of three learning models: “artificial neural network”, “K nearest neighbor” and “support vector machine”; is used to identify attacks and security threats in the subnet corresponding to the controller node. In order to reduce the complexity and computational load imposed on the controller nodes, the learning model deployed in these nodes will only analyze the traffic sent from its sub-network nodes. so, it is possible to prevent network equipment and routers from infecting with malicious codes at the beginning of the sending process, and the malicious node can be easily identified. This process is illustrated with an example in Fig. 3.

To maintain simplicity, it is assumed in Fig. 3 that two nodes are located in the same subnet. Node A sends malicious messages and node B is normal. It is assumed that each of these nodes intends to send a message to the other. As mentioned, all network nodes exchange data through their subnet controller, and this controller checks all the messages sent by the subnet members by a neural network model. In the scenario of Fig. 3, when node A sends a malicious message to the controller, before any processing, the characteristics of the package are extracted and classified by the artificial neural network. If the artificial neural network places the received message in the category of attacks; The message will be blocked and deleted. This condition occurred for the hypothetical sending message from node A to node B. On the other hand, the message sent by node B is detected as normal by the neural network located in the controller, and therefore it is sent to the receiver node A. In the following, the process of detecting attacks based on artificial neural network is explained.

The first step in the process of detecting attacks is the standardization of packet traffic information. To standardize the data, the following actions are performed:

- The nominal characteristics of the traffic flow being processed are numerically valued. For example, the "connection type" attribute can have one of ICMP, UDP, and TCP states, and these values are replaced by numbers one to three.
- The numerical characteristics obtained for the traffic flow are normalized using (4).

After normalizing the traffic flow features, the combination of "artificial neural network", "K nearest neighbor" and "support vector machine" is used to detect attacks through the obtained features. Each of the mentioned learning models is trained independently and using training samples. Then the test samples (network traffic features) are processed by each of these learning models and the output of each model is defined as a logical variable. In this case, the True output for each learning model means there is an attack, and the False output means that the data flowing in the network is normal. After determining the output of the three learning models used in the proposed aggregate system, the voting technique is used to determine the result of intrusion detection. In this case, each test sample will belong to the output class whose label corresponding to that class has the highest vote among the learning models. In other words, the proposed aggregate system will recognize a traffic flow as an attack if at least two learning models in this system detect the characteristics of that traffic flow as an intrusion.

The remainder of this section describes the characteristics of the classifications used in the proposed aggregate system.

#### K nearest neighbor

The K-nearest neighbor method is one of the simplest machine learning algorithms for classification purposes. In this algorithm, a sample is classified by the majority vote of its neighbors and this sample is determined in the most general class among k nearest neighbors. The k-nearest neighbor method is used for many methods because it is effective, non-parametric, and easy to implement. For this reason, in SRAIOT, it is considered as one of the aggregate model algorithms. This algorithm classifies a test sample based on k nearest neighbors. The training samples are represented as vectors in the multidimensional feature space. The space is partitioned into regions with training samples. A point in the space belongs to a class that has the most training points belonging to that class within the closest training sample to k in it^30^. In SRAIOT, the Euclidean distance criterion is used in the KNN model. Also, the parameter K or the number of nearest neighbors is set equal to 5.

#### Support vector machine

The second learning model used in the proposed aggregate system is the support vector machine. Algorithms based on support vector machines are algorithms that try to maximize a margin. To find the categories separating line, these algorithms start from two parallel lines and move these lines in opposite directions so that each line reaches a sample of a specific category on its side. After this step, a strip or border is formed between two parallel lines. The greater the width of this band, it means that the algorithm was able to maximize the margin and the goal is to maximize the margin^31^. The shape of the boundary between the plates separating categories is determined through the kernel function of the support vector machine. In SRAIOT the linear kernel function is used to detect attacks in each subnet.

#### Artificial neural network

**Figure 4:**
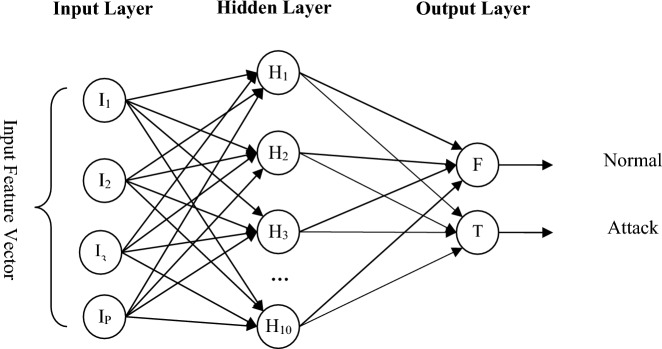
Neural network structure for detecting the presence of attacks in each controller node.

This neural network is a perceptron network with a hidden layer. The hidden layer of this network has 10 neurons and its transfer function is defined as logarithmic sigmoid. Also, the number of neurons in the input layer is equal to the number of features of the traffic flow, and the number of neurons in the output layer is 2. The output value of this neuron indicates the existence of an attack in the network. The structure of this network is shown in Fig. 4. Levenberg–Marquardt backpropagation algorithm^32^ is used to train the neural network. This algorithm performs network learning by bringing the output error closer to zero and based on the Jacobi matrix.

As mentioned, after determining the output of each of the above three learning models in the controller node, voting is done between the outputs and the result of attack detection is based on the result of the majority vote.

## Simulation and results

In this section, the performance evaluation of SRAIOT is performed. The simulation is done in MATLAB software. The distribution of nodes in the network environment is considered to be random and normal. All network nodes are mobile and are not aware of their position in the environment. It is also assumed that each network node has a limited and non-renewable energy source with an initial power of 0.5 J. If the energy of each node runs out, its life will also end and it will not be usable. Also, the noise coefficient of the environment is considered equal to 5 to simulate the amount of fading. The most important parameters used in the simulation environment are presented in Table 2.

**Table 2:** Simulation parameters.

Value	Parameter
500 m * 500 m	Dimensions of the environment
Variable between 100 and 300 nodes	Number of network nodes
Random between 0.5 and 1 J	Initial energy of each node
Random between 50 and 100 KB	Buffer capacity of each node
Variable between 80 and 120 packets per second	maximum packet send rate
0.1 network nodes	Malicious nodes

In order to conduct tests, the performance of SRAIOT is studied in two different scenarios:

- Change in the number of network nodes
- Change in network sending packet rate

The results obtained for SRAIOT in each of these tests are compared with the CLCSR method in^12^ and the DCNN-DPFES method in^13^. In order to reduce the effect of the error caused by random situations, the assays were repeated 20 times and the average of the obtained results is given. In addition to the above two scenarios, the efficiency of the proposed EL model in detecting attacks was studied using the data available in the NSLKDD database, and the accuracy of the proposed system in detecting different types of attacks was checked. In The remainder of this section, the results of the above test scenarios are described and analyzed.

### Performance of SRAIOT for different number of nodes

In this scenario, several nodes are uniformly and randomly deployed in an environment with 500*500 m dimensions. The dimensions of the environment are fixed. Each node in the network environment can be a data source and after generating data, it sends it to the destination node. Also, the number of network nodes in each test changes from 100 to 300. The radio range of each node is set to 100 m and the data rate of the network nodes is 100 kilobits per second.

**Figure 5:**
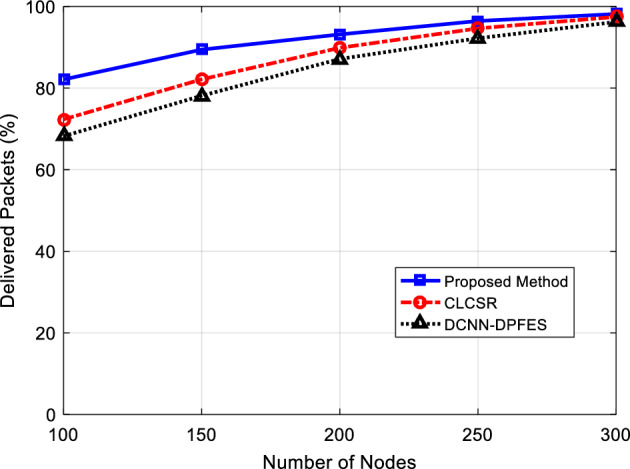
The percentage of successfully delivered packets for different number of nodes.

In order to minimize the error in the simulation, each assay is repeated 20 times and the average of the results is evaluated. The percentage of successful packet reception is especially important for critical and emergency applications where every packet must be received by the destination. This criterion is calculated by dividing the number of successfully sent packets to the destination by the total number of sent packets and is defined as the following relationship:

5 $$Delivery Ratio=100\times \frac{D}{T}$$

where T is the number of packets produced by the source nodes and D is the number of packets received by the destination. The results of this assay are shown in Fig. 5. According to the results, with the increase in the number of network nodes, SRAIOT has a higher packet delivery rate. The upward trend of the displayed graph can be justified by the fact that with the increase in the number of network nodes, the density of the network increases, and as a result, more routes are available to the network nodes, and the routing algorithm can improve the performance of the network in the delivery of data packets by selecting more optimal routes.

The results of the simulation show that SRAIOT works better than compared methods in all situations and has a minimum successful reception of 82% and a maximum reception of 98% for the number of variable nodes. These results show that SRAIOT performs better in selecting routes with less traffic. Also, the packet loss rate in SRAIOT is lower than the packet loss rate in compared methods.

Efficiency in energy consumption can be considered as one of the key parameters in measuring the performance of wireless networks with limited resources. Considering the limited energy resources of network nodes, the efficiency feature in energy consumption is very important. In this section, the energy consumption of the network is evaluated for the number of different nodes.

**Figure 6:**
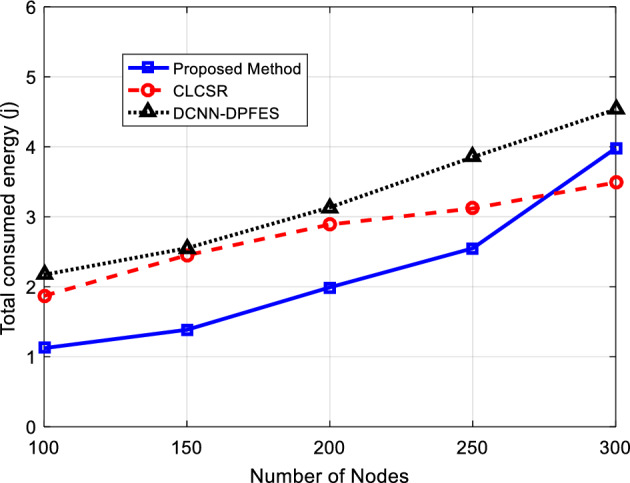
Energy consumption of the whole network for different number of nodes.

Figure 6 shows the energy consumption of all nodes for the number of different nodes in the network. With the increase in the number of network nodes, the number of active connections in the network increases, therefore the amount of energy consumption in the network increases. The results of this test show that the amount of increase in energy consumption in SRAIOT compared to the number of nodes is less than the amount of increase in compared methods and SRAIOT can control the energy consumption in the network more efficiently. The higher efficiency in energy consumption for SRAIOT can be attributed to the selection of paths with more energy for data routing. On the other hand, by increasing the number of network nodes to 300, the energy consumption of SRAIOT has increased compared to the CLCSR method. The reason for this increase is the higher number of packets delivered to the destination. Because in SRAIOT, the packet delivery rate is higher than compared method, and this means that the network nodes use more energy for data routing in the network.

**Figure 7:**
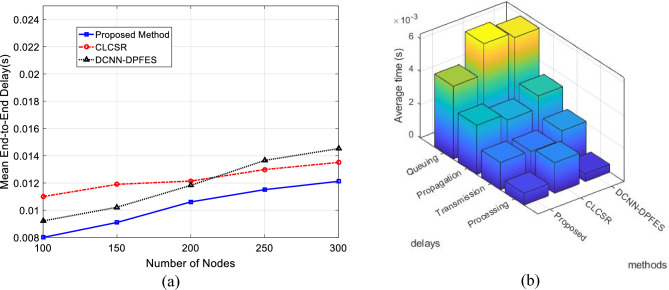
(**a**) Average network end-to-end delay for different number of nodes (**b**) The average end-to-end delay of methods in terms of into propagation, processing, transmission and queueing delays.

Figure 7a shows the graph of the average end-to-end delay for changes in the number of users. The end-to-end delay is calculated as the time interval between sending data by the source and receiving the packet by the destination node. As it is clear from the results of this test, the increase in the number of users increases the end-to-end delay in the network. Because with the increase in the number of users, the number of clusters and, as a result, the number of steps required to route data to the destination increases. Nevertheless, using the parameters of congestion degree and distance as criteria for determining the weight of network connections and forming a hierarchical topology based on these criteria causes SRAIOT to reduce the end-to-end delay. Also, Fig. 7b, decomposes the average end-to-end delay of each method into propagation, processing, transmission and queueing delays. As shown in Fig. 7b, the queuing delay of the proposed method is significantly lower than compared methods which is the result of utilizing a mobility-based hierarchical topology. On the other hand, the difference between propagation and transmission delays in proposed method is fewer than other methods which means transmitted packets are less likely to be dropped during routing.

**Figure 8:**
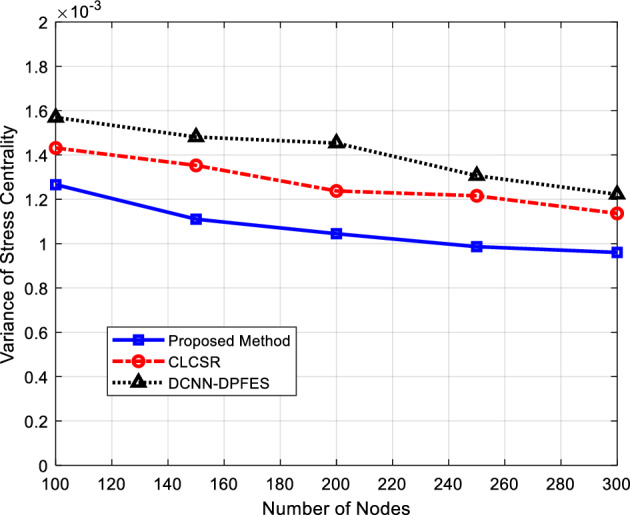
Variance of stress centrality on links for different number of nodes.

In Fig. 8, the variance of stress centrality on links for different routing methods have been compared. Stress centrality is a metric for describing the behavior of routing algorithms in distributing traffic load on the network. Stress centrality on a link can be formulated as^33^:

6 $$Stress\left(L\right)=\sum_{i\ne j\in V}{\delta }_{ij}(L)$$

where L is the desired ink for evaluating stress centrality. Also, i and j are two nodes in the network. Finally, ${\delta }_{ij}(L)$ is a binary function which would be equal to 1, if there exists a route between I and j which includes link L; otherwise it will be zero.

Stress centrality measures the traffic load per link. As shown in Fig. 8, Increasing the number of network nodes will cause a slight decrease in stress variance. Because with the increase in the number of nodes, the degree of connection between network nodes and the number of links between them will increase on average, and this will mean the possibility of establishing a more coordinated load distribution between network links by each algorithm. The results presented in this experiment show that the proposed method can work more successfully in reducing stress variance. Because the topology formation process in the proposed method by using the hierarchical structure can, in addition to controlling the degree of nodes, select a subset of network connections that distribute network traffic in a more balanced way.

### Performance of SRAIOT for different data rate

**Figure 9:**
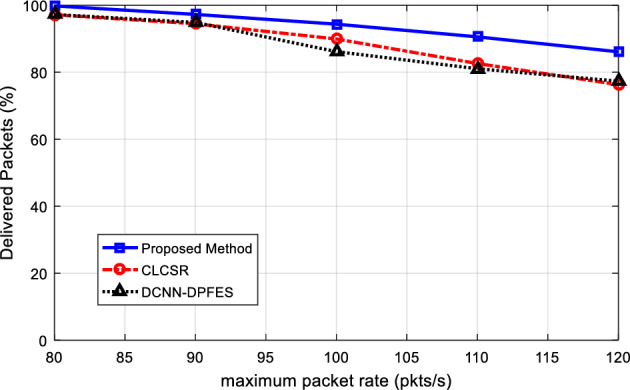
The percentage of successfully delivered packets for different network data rate.

A suitable routing algorithm should be able to perform well at high data rates. To investigate this feature in SRAIOT, the effect of increasing the data rate is investigated. In this test, the number of network nodes is set equal to 100 nodes and the data rate of network nodes is changed from 80 to 120 packets per second. In Fig. 9, the graph of the change in the successful delivery rate depending on the change in the data rate is displayed.

As the network data rate increases, the number of packets being exchanged in the network increases at every moment. This will increase network traffic and thus increase the possibility of packet loss. As the results in Fig. 9 illustrate, SRAIOT is more resistant to increasing the data rate and, in all cases, has a higher percentage of successful delivery than compared methods.

**Figure 10:**
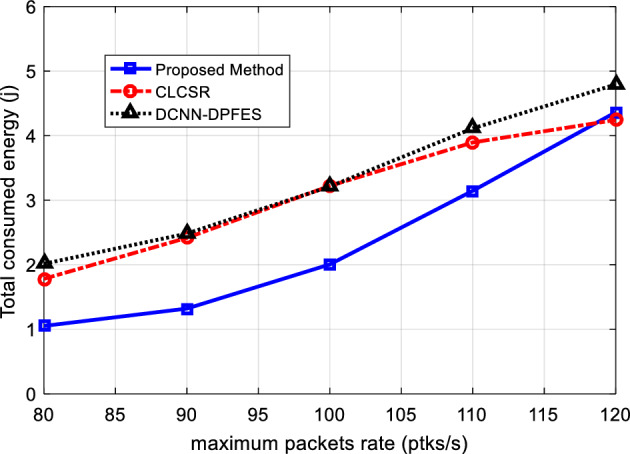
Total network energy consumption for different network data rate.

The graph of energy consumption of all network nodes in this test is shown in Fig. 10. Based on the simulation results, SRAIOT in most cases has less energy consumption. This result shows that SRAIOT works well in selecting routes that cause less energy consumption. The reason for this improvement can be attributed to considering the characteristics of distance and energy for weighting network connections. Because considering these criteria to form the network's hierarchical topology structure will lead to the formation of paths for data transmission so that its middle nodes have more residual energy in addition to less distance. As a result, less energy is required for data exchange, which is obvious in the presented results.

**Figure 11:**
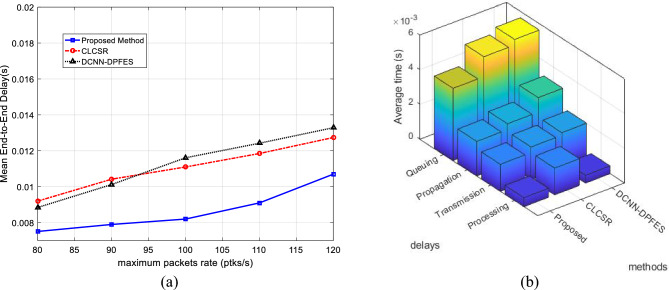
(**a**) Average network end-to-end delay for different network data rate (**b**) The average end-to-end delay of methods in terms of into propagation, processing, transmission and queueing delays.

Figure 11a shows the graph of the average end-to-end delay for changes in the maximum data rate. Based on these results, increasing the data rate will increase the delay. Because with the increase of data rate, network traffic increases and the possibility of congestion in network nodes increases; As a result, each data packet will be in the network for a longer time and the end-to-end delay will also increase. However, considering the hierarchical tree topology structure in SRAIOT has simplified the communication structure of the network. In addition to increasing the scalability of the network, this structure has made it possible to maintain the proper performance of the network even in conditions of increased traffic. On the other hand, considering the movement pattern of nodes to form network sub-domains leads to the formation of more stable connections in the network. Because SRAIOT guarantees the stability of connection for a minimum period of Δt. As a result, the possibility of sudden deletion of a connection link and the need to resend the packet through secondary routes will be negligible. These characteristics cause SRAIOT to have a lower average delay.

Figure 11b shows that the proposed method can work more successfully than the compared methods in terms of controlling each of the delay components. The lower queuing delay in the proposed method shows the quick response of the routing algorithm and its efficiency in preventing packets from waiting during the routing process. On the other hand, the lower processing delay compared to other methods confirms the computational efficiency of this method. These results show that the proposed routing model can maintain its optimal performance in the conditions of increased traffic load.

### Performance of SRAIOT in detecting attacks

As explained in the third part, in SRAIOT, EL models based on the controller nodes are used to detect attacks in the traffic flowing in the network. In this section, the effectiveness of the proposed model is checked using real network traffic data. For this purpose, the proposed model has been tested using the data available in the NSLKDD database^34^. The database includes more than 25,000 data records in the field of information exchanged in the network. These records contain the information of the packets sent during the occurrence of various network intrusions. The information available in the NSLKDD database is shown in Table 3.

**Table 3:** Types of information available in the NSLKDD database ^34^.

Percentage of data available (%)	Type of attacks
19.48	Normal connection (no attack)
73.9	DoS attacks
1.34	U2R attacks
5.2	R2L attacks
0.07	Probe attacks

As shown in Table 3, about 80% of the information in the database is dedicated to attacks. Each data record in this database contains 42 statistical features of traffic flow characteristics. In the simulation process, it is assumed that each attacking node in the network exchanges one of the data records in this database with other nodes. During the data exchange process, the data flow traffic information is classified by the aggregate model available in the controller node and the presence of attacks is detected. In the following, the performance of the proposed aggregate system in separating normal traffic flows from the flows related to attacks is examined.

As mentioned at the beginning of this section, the simulation operation was repeated 20 times. During this process, in each repetition, 95% of database samples are used as training samples of learning models and the remaining 5% are used as test samples. The selection of training and test samples in each iteration is done randomly. Also, the effectiveness of the proposed ensemble system in detecting attacks with the condition that its classifications are used independently to detect attacks; is compared. Also the proposed ensemble system has been compared with attack detection methods proposed in^27^ and^28^.

**Figure 12:**
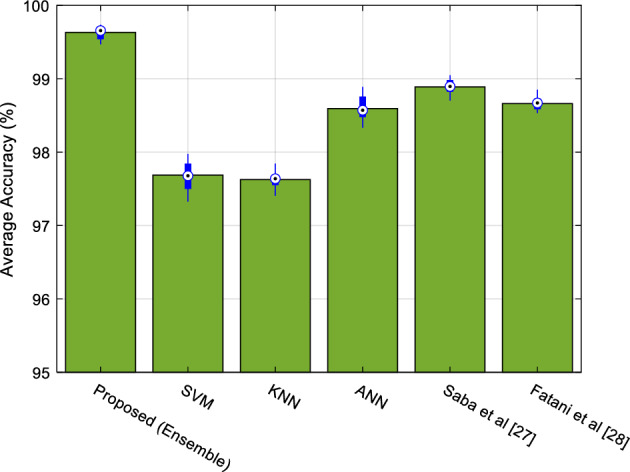
The accuracy of learning algorithms in detecting network attacks by the controller node.

The accuracy results of each of the compared algorithms in detecting network attacks are shown in Fig. 12.

As it is clear from the results shown in Fig. 12, by using the proposed ensemble system, it is possible to detect attacks with an accuracy of 99.6%, which shows an improvement of at least one percent in accuracy compared to each classifier which forms this model. The increase in detection accuracy in the proposed system can be seen as the result of using the ensemble technique. Because in a EL system, learning models can cover minor errors in attack detection through sharing their knowledge. On the other hand, compared to the methods^27^ and^28^, the proposed method has an advantage of 0.75 and 0.97%, respectively, which shows the effectiveness of the simple ensemble technique in improving the performance of machine learning models.

**Figure 13:**
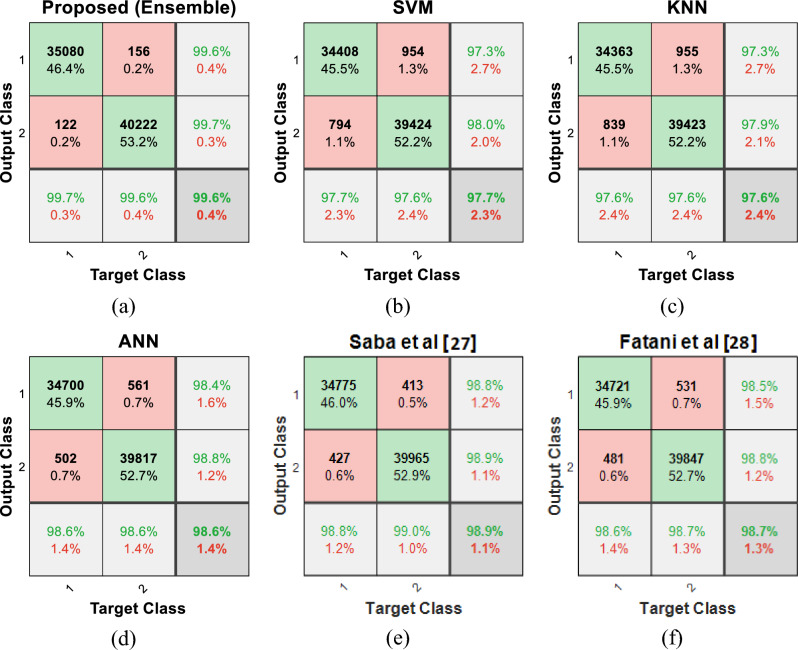
Confusion matrix of the (**a**) proposed ensemble system (**b**) SVM, (**c**) KNN, (**d**) ANN, (**e**) Saba et al. ^27^ and (**f**) Fatani et al. ^28^ for classifying network traffic flows.

In order to more closely examine the performance of the proposed ensemble system in identifying attacks, the confusion matrix is examined. Figure 13a shows the confusion matrix resulting from the detection of network attacks during 20 iterations of the simulation for SRAIOT. In the displayed confusion matrix, the number 35080 located in the first row and column indicates the number of tested normal packages that are correctly recognized as normal by the proposed model. This number is identified as TN in the confusion matrix. The number 122, located in the second row and the first column, indicates the number of tested normal packets that were wrongly detected as attacks by the proposed model. This number is identified as FP in the confusion matrix. The second row and the second column of the confusion matrix, which displays the number 40222, indicate the packets related to the attack that are correctly classified by the proposed model and identified as TP samples. Also, the number 156, located in the first row and the second column, indicates the packets related to the attack that were mistakenly recognized as normal by the proposed model. This number is identified as FN in the confusion matrix. These results show that only 0.2% of the infected packets rerouted in the simulation process could pass through the intrusion detection system of the controller nodes. Also, the false alarm rate of the proposed model is 0.002 (122 out of 75.5K samples) which is at least 75% lower that compared methods. This is even though, in SRAIOT, only the outgoing traffic of each subdomain passes through the filter of the intrusion detection system. If this monitoring is applied to both directions of traffic flows, the false positive and false negative rates can be brought closer to zero. Also, the confusion matrices of other methods have been presented in Fig. 13b–f. Comparing the results of the proposed method with these methods shows that SRAIOT works more accurately both in detecting the flow of attacks and in identifying the normal traffic flows of the network.

Table 4 compares the results of testing the proposed model to detect attacks in the network with the results of other learning models. In this table, the sensitivity and specificity criteria are compared. The sensitivity criterion is used to measure the total proportion of attacks that are correctly detected by the learning model and is calculated as follows^35^:

**Table 4:** Comparing the efficiency of the proposed model with other learning models.

Algorithm	Sensitivity	Specificity	Accuracy (%)
SVM	0.9764	0.9774	97.6872
KNN	0.9763	0.9761	97.6264
ANN	0.9861	0.9857	98.5935
Saba et al. ^27^	0.9897	0.9878	98.8886
Fatani et al. ^28^	0.9868	0.9863	98.6610
Proposed (Ensemble)	0.9961	0.9965	99.6322

7 $$Sensitivity=\frac{TP}{TP+FN}$$

where, TP is the number of attack flows that are correctly detected and FN is the number of attack traffic flows that are wrongly detected as normal flows.

The Specificity criterion is used to measure normal flows that are correctly classified. This criterion is calculated as follows^35^:

8 $$Specificity=\frac{TN}{TN+FP}$$

where TN is the number of normal traffic flows that are correctly detected, and FP is the number of normal traffic flows that are detected as attack traffic flows.

**Figure 14:**
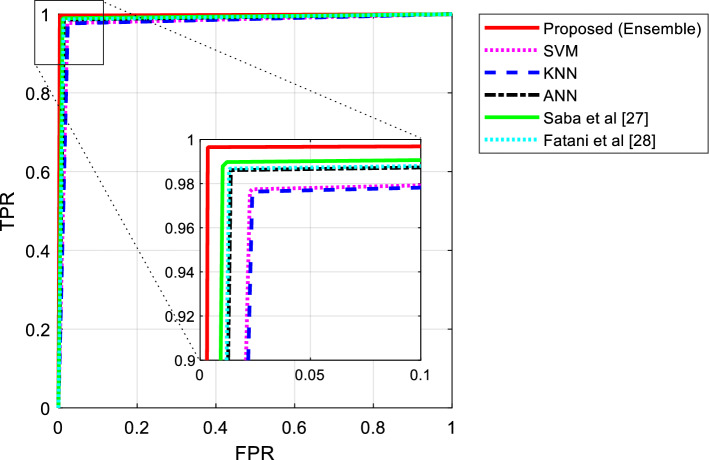
The ROC curve of different method in classifying network traffics.

The results of the execution of this scenario showed that by using the proposed aggregate model, the presence of attacks in the network could be done with higher accuracy. In this case, in addition to increasing the accuracy, the voting strategy can also perform well in increasing sensitivity and specificity. Thus, the proposed model can be effective both in detecting normal traffic flows and in detecting network attacks more accurately. Figure 14, presents the Receiver Operating Characteristics (ROC) curve of different methods in classifying network traffic flows.

This figure, shows the performance of each learning model in different classification thresholds. As shown in Fig. 14, the attack detection by SRAIOT results in reduced false positive rates and higher true positive rate values. Therefore, the Area Under the Curve (AUC) of the proposed method is higher than compared methods. These results, prove the superiority of the propose method in more accurate detection of network attacks.

## Conclusion

In this article, a new and efficient method for secure and efficient data routing with the ability to identify attackers in the IoT was presented. SRAIOT uses the clustering solution based on the movement pattern of active nodes to divide the network topology into several subdomains. In this structure, each section is equipped with a controller to exchange security rules with other subdomains, and all the traffic of nodes related to a subnet is exchanged through the controller node of that subnet. Therefore, each controller node continuously uses an EL model to analyze network traffic information and identify attacks. The use of this integrated system, according to its position, makes it possible to analyze the network traffic at a lower cost and, on the other hand, to increase the accuracy of network intrusion detection by using several learning models. In order to implement and evaluate SRAIOT, its performance was studied from two aspects: routing and the ability to detect attacks. The results of these tests showed that the structure presented in SRAIOT improves the network performance during the routing process and can effectively reduce energy consumption and end-to-end delay, furthermore can increase the packet delivery rate. In addition, the proposed aggregate model can detect the presence of attacks in network traffic flows with 99.6 percent accuracy, and these results show values ​​of 99.67 and 99.59 for the sensitivity and specificity criteria. All these features make SRAIOT a suitable and reliable solution for secure data routing in real-time IoT applications.

This research used SDN as an infrastructure for deploying the security model. This is while the SDN architecture itself has security issues that need to be addressed. For example, the biggest security issue about SDN is the communication channel. Because, the OpenFlow protocol, uses Transport Layer Security (TLS) for data-control channel communication security, and it is prone to man-in-the-middle attacks. Addressing these issues requires the use of security strategies that are outside the scope of this research; But solving them in order to guarantee the performance of SDN-based security models is of great importance. For this reason, solving the security issues related to the communication channel in SDN can be a topic for continuing this research path. Also, increasing the computational load of the ensemble system in detecting attacks is another limitation of the current research, which results from the use of multiple learning models. Although this increase in computational load is not very noticeable in the detection phase, this limitation can be overcome by using parallel processing techniques or tiny machine learning methods. Therefore, trying to reduce the computational overhead in the proposed intrusion detection component can be the subject of future research works.

## Data Availability

All data generated or analyzed during this study are included in this published article.
